# Supplementary material for: Advancing clinical cohort selection with genomics analysis on a distributed platform
Source: PLoS One. 2020 Apr 23;15(4):e0231826. doi: 10.1371/journal.pone.0231826 (PMC7179830; doi:10.1371/journal.pone.0231826)
Supplement: S1 Repository — https://github.com/GenomicsDB/GenomicsDB. (DOCX) [file pone.0231826.s001.docx]

**S1 Repository. GenomicsDB.** <https://github.com/GenomicsDB/GenomicsDB>.
