# Supplementary material for: Advancing clinical cohort selection with genomics analysis on a distributed platform
Source: PLoS One. 2020 Apr 23;15(4):e0231826. doi: 10.1371/journal.pone.0231826 (PMC7179830; doi:10.1371/journal.pone.0231826)
Supplement: S3 Repository — 10.6084/m9.figshare.11796126. (DOCX) [file pone.0231826.s003.docx]

**S3 Repository. Raw runtimes.** [10.6084/m9.figshare.11796126](https://doi.org/10.6084/m9.figshare.11796126).
