# Supplementary material for: Advancing clinical cohort selection with genomics analysis on a distributed platform
Source: PLoS One. 2020 Apr 23;15(4):e0231826. doi: 10.1371/journal.pone.0231826 (PMC7179830; doi:10.1371/journal.pone.0231826)
Supplement: Supplementary file 3 [file pone.0231826.s002.docx]

S2 Repository. VariantView Plugin. https://github.com/OmicsDataAutomation/i[illegible]
