# Supplementary material for: Advancing clinical cohort selection with genomics analysis on a distributed platform
Source: PLoS One. 2020 Apr 23;15(4):e0231826. doi: 10.1371/journal.pone.0231826 (PMC7179830; doi:10.1371/journal.pone.0231826)
Supplement: Attachment — Submitted filename: Response To Reviewers.docx [file pone.0231826.s004.docx]

Jianjiong Gao

Academic Editor

PLOS ONE

Dear Dr. Gao,

Thank you for facilitating the prompt review of our manuscript entitled “Advancing Clinical Cohort Selection with Genomics Analysis on a Distributed Platform” by Smith et al, that was submitted for consideration in the digital heath call for papers. We were very glad that you and the reviewers felt our work represented an important technical advance. Using reviewer feedback as a guide, we have made further improvements to the manuscript that we have itemized as follows:

Reviewer 1. Overall comments were very positive. Reviewer 1 did include a short list of suggested “minor adjustments that may benefit the paper”. We respond to them in order as follows:

1) Suggested clarification regarding the log-scale potential of microarray data and how these results could expand into whole genome sequencing datasets.

   This is an important point, and we have extended the discussion section (page 24) to reflect this. In summary, the results suggest strong correlation between import and query time and the amount of data involved. Smaller data sizes will suffer more from start up costs without much gain, whereas the larger datasets are able to take more advantage of the distributed processing environment. Given the microarray data starts to show linear behavior for the largest query region, and that the much heavier, whole exome data continues to show linear behavior for this query, suggests that this trend will continue for heavier, whole genome data.
2) Suggested including specific queries run with the I2B2 plugin module.

We have extended the introduction (pages 5,6) with I2B2 workflow examples that can be used as motivating examples. In addition, the experiments section now describes further clinical filters that were explored (page 21), and provides an explanation for the types of genomic regions considered (page 19).

3) Making code for the I2B2 plugin and the GDBSparkAPI available for academic use was highly encouraged.

We very much agree with this sentiment. Facilitating implementation and use of these tools in a generalizable way in academic settings, has been a goal of this project from its inception. With this in mind, we have made a version of the i2b2 plugin available under the MIT license, and the GDBSparkAPI and gdb-mapping database available for academic use. These components can be found at https://github.com/OmicsDataAutomation/i2b2-oda-framework. All GenomicsDB components are available at: https://github.com/GenomicsDB/GenomicsDB for free academic use under the MIT license. I2B2 patient querying related components are available at https://www.I2B2.org/software/index.html.

Reviewer 2. Overall comments were again very positive. This reviewer also requested example queries demonstrating the potential utility of these tools to researchers. We address this in item 2) above.

Once again, thank you for the prompt and thorough reviews. We very much hope that you now find our revised manuscript to be suitable for publication in PLOS ONE. We look forward to hearing from you at your earliest convenience.

Sincerely,

Jaclyn Smith

DPhil Candidate

Department of Computer Science, University of Oxford

Principal Engineer

Omics Data Automation
